# Quality of life after traumatic brain injury: a cross-sectional analysis uncovers age- and sex-related differences over the adult life span

**DOI:** 10.1007/s11357-020-00273-2

**Published:** 2020-10-17

**Authors:** Katrin Rauen, Claudia B. Späni, Maria Carmela Tartaglia, Maria Teresa Ferretti, Lara Reichelt, Philipp Probst, Barbara Schäpers, Friedemann Müller, Klaus Jahn, Nikolaus Plesnila

**Affiliations:** 1 Schoen Clinic Bad Aibling, Kolbermoorer Strasse 72, 83043 Bad Aibling, Germany; 2 Institute for Stroke and Dementia Research (ISD), University of Munich Medical Center, Feodor-Lynen-Strasse 17, 81377 Munich, Germany; 3 Department of Geriatric Psychiatry, University Hospital of Psychiatry Zurich, Minervastrasse 145, 8032 Zurich, Switzerland; 4 Institute for Regenerative Medicine (IREM), University of Zurich, Wagistrasse 12, 8952 Schlieren, Switzerland; 5 Women’s Brain Project, Pfisterwisstrasse 14, 8357 Guntershausen, Switzerland; 6 Feinberg School of Medicine, Department of Physiology, Northwestern University, 310 East Superior St., Chicago, IL 60611 USA; 7 Tanz Centre for Research in Neurodegenerative Diseases, University of Toronto, 60 Leonard Ave, 6KDT407, Toronto, ON M5T 2S8 Canada; 8 Institute for Medical Informatics, Biometry and Epidemiology (IBE), Marchioninistr 15, 81377 Munich, Germany; 9 German Center for Vertigo and Balance Disorders, University of Munich Medical Center, Marchioninistrasse 15, 81377 Munich, Germany; 10 Munich Cluster for Systems Neurology (Synergy), Munich, Germany

**Keywords:** Aging, Health-related quality of life, QOLIBRI, Psychiatric disorders, Age- and sex-related outcomes, Traumatic brain injury

## Abstract

Traumatic brain injury (TBI) is the leading cause of disability in the working population and becomes increasingly prevalent in the elderly. Thus, TBI is a major global health burden. However, age- and sex-related long-term outcome regarding patient’s health-related quality of life (HRQoL) is yet not clarified. In this cross-sectional study, we present age- and sex-related demographics and HRQoL up to 10 years after TBI using the Quality of Life after Brain Injury (QOLIBRI) instrument. The QOLIBRI total score ranges from zero to 100 indicating good (≥ 60), moderate (40–59) or unfavorable (< 40) HRQoL. Two-thirds of the entire chronic TBI cohort (102 males; 33 females) aged 18–85 years reported good HRQoL up to 10 years after TBI. TBI etiology differed between sexes with females suffering more often from traffic- than fall-related TBI (*p* = 0.01) with increasing prevalence during aging (*p* = < 0.001). HRQoL (good/moderate/unfavorable) differed between sexes (*p* < 0.0001) with 17% more females reporting moderate outcome (*p* = 0.01). Specifically, older females (54–76-years at TBI) were affected, while males constantly reported good HRQoL (*p* = 0.017). Cognition (*p* = 0.014), self-perception (*p* = 0.009), and emotions (*p* = 0.016) rather than physical problems (*p* = 0.1) constrained older females’ HRQoL after TBI. Experiencing TBI during aging does not influence HRQoL outcome in males but females suggesting that female brains cope less well with a traumatic injury during aging. Therefore, older females need long-term follow-ups after TBI to detect neuropsychiatric sequels that restrict their quality of life. Further investigations are necessary to uncover the mechanisms of this so far unknown phenomenon.

**Electronic supplementary material:**

The online version of this article (10.1007/s11357-020-00273-2) contains supplementary material, which is available to authorized users.

## Introduction

Traumatic brain injury (TBI) is the number one cause of mortality among children and young adults and becomes increasingly prevalent among the elderly [1]. In Europe, 2.5 million people suffer a TBI each year with an annual hospital discharge rate of 287 per 100,000 people [2, 3]. Thus, TBI is a major, health, and socio-economic burden at the personal and global level with 247.6 Mill. disability-adjusted life years (DALY) between 1990 and 2013 [4, 5]. The male to female ratio in TBI ranges up to 3–4:1, and young males are believed to be more prone to severe TBI due to a pronounced risk behavior specifically during sportive activities [6, 7]. Later in life, the etiology of TBI changes from sports and road traffic accidents to falls at home [1, 8].

For a long time, TBI outcome research has mainly focused on the patient’s physical performance using the Extended Glasgow Outcome Scale (GOSE) at 6 and 12 months post-TBI. However, when assessing the TBI long-term outcome beyond 12 months, health-related quality of life (HRQoL) is thought to be more suitable since it integrates more relevant outcome measures than merely physical function [1]. The World Health Organization (WHO) defines quality of life as “an individual’s perception of their position in life in the context of the culture and value systems in which they live and in relation to their goals, expectations, standards and concerns….” [9]. Thus, TBI outcome is influenced not only by the brain injury itself but also by the individual’s sex- and gender-related experiences which play a major role for the long-term outcome after TBI [10].

The Quality of Life after Brain Injury (QOLIBRI) instrument is a disease-specific, health-related, and internationally validated instrument to assess quality of life after brain injury. The QOLIBRI covers six subscales including cognition, self (including, e.g., energy, motivation, self-esteem, self-perception), daily life and autonomy, social relationships, emotions, and physical problems, thereby reflecting the patient’s well-being. For its validation, the QOLIBRI instrument has been systematically related to the patient’s emotional state, functional outcome, comorbidities, and generic health using the Hospital Anxiety and Depression Scales, the GOSE, a health questionnaire regarding 28 comorbidities, and the Short Form health survey (SF-36) [11, 12], thereby making it a most suitable and specific TBI outcome measure.

In most chronic TBI studies, female patients make up one-third of the sample and the studies did not stratify for sex- and age-related HRQoL when using the QOLIBRI instrument [12–17]. Nevertheless, two large chronic TBI outcome studies on HRQoL did not find sex-related differences in patients aged 17 to 69 years up to 15 years after TBI when using the QOLIBRI total score or its overall scale [11, 18]. Bearing in mind that reporting sex and gender differences in brain research becomes increasingly important, “sex” refers to all biological factors and “gender” to all identity, psychosocial, cultural, or socio-economic aspects [19]. Gender-related diversity in education, professional challenges, income, as well as gaps in working periods lead to gender effects in general and might influence women’s posttraumatic mental health and HRQoL—factors that are understudied in brain research. Furthermore, it is currently unclear why translational TBI research has not yet been successful. The heterogeneity of TBI and age- and sex-related differences of brain vulnerability might be additional factors that hamper successful translation. Thus, the aim of this cross-sectional analysis was to highlight age- and sex-related HRQoL up to 10 years after TBI over the adult lifespan assessing patients aged 18 to 85 years.

## Methods

### Study design

In this cross-sectional study, we analyzed sex- and age-related differences of HRQoL in 135 out of 439 consecutively admitted TBI patients who received primary care within a hospital of the Southern Upper-Bavaria Trauma Network followed by standardized neurorehabilitation at Schoen Rehabilitation Center, Bad Aibling, Germany, between 2005 and 2015. This chronic TBI cohort, from now on named QOLIBRI cohort, received comparable medical and rehabilitative care as previously described [20, 21]. In November 2015, 439 TBI patients were invited by letter to participate in a quality of life study using the QOLIBRI questionnaire up to 10 years after discharge from Schoen Rehabilitation Center. In cases with severe cognitive and/or motor impairment, the patient’s caregiver helped in completing the questionnaire. According to the local legislation of the Bavarian Hospital Law (BayKrG) and the ethical committee of the Ludwig-Maximilians University, Munich, Germany, ethics approval was not required for this study.

### Sex-related demographic and basic characteristics

Sex differences (male/female) were quantified for (i) each TBI severity group (mild, moderate or severe) (%), (ii) TBI etiology (traffic accident, fall or others) (%), (iii) the age at TBI (mean ± SEM), (iv) the age at the time of the survey (mean ± SEM), (v) the elapsed time since TBI in years (mean ± SEM), (vi) whether a decompressive craniectomy (DC) was performed (%), (vii) whether an intracranial pressure (ICP) probe or a permanent shunt device was implanted (%), (viii) whether a tracheostomy was performed (%), (ix) the time to onset of neurorehabilitation (days ± SEM), (x) the duration of neurorehabilitation (days ± SEM), (xi) the functional status at admission (mobile/immobile), and (xii) the functional status at discharge from neurorehabilitation (mobile/immobile). Functional status was continuously analyzed as well as dichotomized; patients were assigned mobile with a modified Rankin Scale (mRS) of 0–3 or immobile with a mRS ≥ 4. TBI severity level was classified using the established categories: mild (TBI I°: GCS 13–15), moderate (TBI II°: GCS 9–12), or severe (TBI III°: GCS 3–8), based on the initially documented score on the Glasgow Coma Scale (GCS) [22], which was extracted from the referral letter to neurorehabilitation. The parameter *time since TBI* describes the elapsed years between the TBI and the assessment of the patient’s HRQoL. Sex-related demographic and baseline characteristics are given for the QOLIBRI cohort and non-responders as well as for the subgroup of 54–76-year-old male and female responders.

### The QOLIBRI instrument

The QOLIBRI instrument is a health-related, disease-specific, and internationally validated instrument to assess HRQoL in patients after brain injury [11, 12]. The QOLIBRI instrument is free to use for researchers, clinicians, and non-profit organizations after registration on the webpage (https://qolibrinet.com/) and consists of the QOLIBRI total score; two major key aspects *satisfaction* and *bothered*, i.e., restrictions; and six subscales (cognition, self, daily life and autonomy, social relationships, emotions, physical problems) [23]. *Satisfaction* is a sum score of cognition, self, daily life and autonomy, and social relationships with the score ranging from 0 to 400, while *restrictions* is a sum score of emotions, and physical problems ranging from 0 to 200. The QOLIBRI total score and the score of the six subscales range from zero to 100, representing lowest and highest HRQoL, respectively. A QOLIBRI total score of ≥ 60 represents good, a score < 60 indicates unsatisfied HRQoL, and the latter can be further distinguished as follows: a score of 40–59 represents moderate HRQoL with an increased risk of one psychiatric disorder, either depressive or anxiety disorder, and a score < 40 represents unfavorable HRQoL with the risk of dual psychiatric disorders [11, 12].

### Sex- and age-related HRQoL up to 10 years after TBI

TBI outcome and HRQoL (QOLIBRI total score) were compared between male and female TBI patients. Sex-related distribution and dichotomized analysis of good (QOLIBRI total score ≥ 60) versus unsatisfied (QOLIBRI total score < 60) HRQoL are given. Sex-related HRQoL (QOLIBRI total score, key aspects, six subscales) was investigated for a subgroup of patients aged 54–76 years at TBI by a post hoc analysis as females of this age group revealed moderate outcome. Furthermore, HRQoL (QOLIBRI total score) was correlated to TBI severity (GCS) for males and females of the QOLIBRI cohort and for the subgroup of the 54–76-year-olds at TBI.

### Data management

All QOLIBRI questionnaires were checked for completeness. Each QOLIBRI responder was assigned an interim ID number. The QOLIBRI scores were added to the demographic and basic characteristics which were obtained from the medical records. Thereafter, the entire data set was anonymized as previously described [20].

### Statistical analysis

Sex- and age-related demographic and basic characteristics of the parameters i–xii were analyzed using the Fisher test for categorical and the Mann-Whitney *U* test for numerical data. All data sets were tested for normal distribution using the D’Agostino-Pearson (omnibus K2) test. Group differences were analyzed by unpaired *t* test for parametric or Mann-Whitney *U* test for non-parametric data. Subgroups of good (QOLIBRI total score ≥ 60), moderate (QOLIBRI total score 40–59), or unfavorable (QOLIBRI total score < 40) HRQoL were analyzed between males and females using the Kruskal-Wallis test. Sex-related dichotomized analysis between good (QOLIBRI total score ≥ 60) and unsatisfied (QOLIBRI total score < 60) HRQoL was performed using the Fisher test. The LOWESS function (Locally Weighted Scatterplot Smoothing) was used to graphically present sex-related HRQoL (QOLIBRI total score) over the adult lifespan. Due to the uncovered sex-related differences in the 54–76-year-olds at TBI, a post hoc analysis of HRQoL (QOLIBRI total score, QOLIBRI key aspects and six subscales) was performed comparing males and females of this age subgroup. Sex-related correlation analyses on TBI severity (GCS) and HRQoL for the QOLIBRI cohort and the subgroup of the 54–76-year-olds at TBI were done for those patients with documented GCS using the Spearman’s rank correlation. Statistical and graphical analyses were performed using R (R Core Team, Vienna, Austria) and GraphPad Prism 8 software (San Diego, CA, USA). Data are reported as relative frequency (%) (TBI severity, TBI etiology, decompressive craniectomy, ICP monitoring/permanent shunt device, tracheostomy, QOLIBRI total score), the mean ± SEM (age at TBI, age at survey, elapsed time since TBI, time to onset of neurorehabilitation, duration of neurorehabilitation, all QOLIBRI scores), and the median with interquartile range (IQR_25–75_) (QOLIBRI total score). Differences or correlations were considered significant at *p* < 0.05. Effect size was assessed using eta squared indicating a small (*η*^2^ ≥ 0.01), medium (*η*^2^ ≥ 0.06), or large effect (*η*^2^ ≥ 0.14) [24].

## Results

**Fig. 1:**
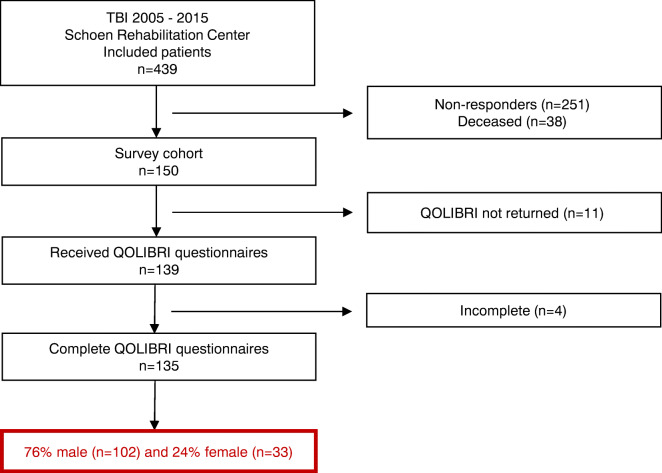
Flowchart depicting chronic TBI patients. In this cross-sectional study, 102 male (76%) and 33 female (24%) adult TBI patients reported their HRQoL up to 10 years after neurorehabilitation. QOLIBRI, Quality of Life after Brain Injury; TBI, traumatic brain injury

In this cross-sectional study, 102 male (76%) and 33 female (24%) adult chronic TBI patients reported their HRQoL up to 10 years after neurorehabilitation due to a TBI (Fig. 1). Most demographic and basic characteristics did not differ between males and females of the QOLIBRI cohort (Table 1), except TBI severity and etiology. In detail, TBI severity classified by the initial GCS differed between males and females with 13% more male than female patients within the category of initially mildly brain injured (*p* = 0.04), but the degree of disability, i.e., the functional status at admission (*p* = 0.32) and discharge (*p* = 0.98) from neurorehabilitation, did not differ between males and females. In detail, male and female patients were severely disabled when admitted to neurorehabilitation with a mean (± SEM) mRS of 4.66 ± 0.07 and 4.46 ± 0.18 (*p* = 0.32), respectively, indicating secondary deterioration after the brain impact. Patients of both sexes recovered during neurorehabilitation and became independent in daily activities but were unable to carry out all previous activities so remained slightly disabled with a mean mRS of 2.25 ± 0.12 in males and 2.3 ± 0.23 in females (*p* = 0.98). Females of our QOLIBRI cohort experienced more often a TBI due to a traffic accident (72.7%) than a fall (24.2%), while males experienced almost equally traffic accidents (43.1%) or falls (46.1%) (*p* = 0.01).

**Table 1:** Sex-related demographic and basic characteristics. Sex-related demographics and basic characteristics are provided for 102 male and 33 female TBI patients of the QOLIBRI cohort. TBI severity was classified according to the initial Glasgow Coma Scale taken from the medical referral letter to neurorehabilitation and revealed more females with severe TBI. However, this difference does not confound current results due to severe disability at admission to neurorehabilitation with no differences between males and females (*p* = 0.32). Why TBI etiology differed between males and females (*p* = 0.01) in the QOLIBRI cohort, with female patients having experienced more often a traffic accident than males, is still not clear, yet. The parameter *time since TBI* describes the elapsed years between the TBI and the assessment of the patient´s health-related quality of life outcome.QOLIBRI, Quality of Life after Brain Injury; TBI, traumatic brain injury.

	Male, *n* = 102	Female, *n* = 33	*p* value
TBI severity mild/moderate/severe/n.s. (%)^a^	16.7/13.7/32.4/37.2	3/12.1/45.5/39.4	0.04*
TBI etiology traffic accidents/falls/others (%)^b^	43.1/46.1/10.8	72.7/24.2/3.1	0.01*
Age at TBI in years (mean ± SEM)^a^	47.41 ± 1.98	47.76 ± 3.36	0.92
Age at survey in years (mean ± SEM)^a^	53.08 ± 1.92	53.24 ± 3.19	0.98
Time since TBI in years (mean ± SEM)^a^	5.71 ± 0.3	5.48 ± 0.53	0.73
Decompressive craniectomy (%)^b^	24.5	36.4	0.19
ICP monitoring or permanent shunt device (%)^b^	55.9	60.6	0.69
Tracheostomy (%)^b^	54.9	51.5	0.84
Time to onset of neurorehabilitation^a^ (days, mean ± SEM)	26.78 ± 2.89	25.61 ± 3.22	0.79
Duration of neurorehabilitation a (days, mean ± SEM)	36.68 ± 2.81	46.09 ± 7.81	0.68
Functional status at admission (mobile (mRS 0–3)/immobile (mRS 4–5)/n.c.(%))^b^ (mean ± SEM)^a^	3.9/96.1 4.66 ± 0.07	9.1/90.9/0 4.46 ± 0.18	0.36 0.32
Functional status at discharge (mobile (mRS 0–3)/immobile (mRS 4–5)/n.c.(%))^b^ (mean ± SEM)^a^	82.4/17.6 2.25 ± 0.12	75.8/24.2/02.3 ± 0.23	0.45 0.98

*n.c.* not classified in the medical record, *n.s.* not specified in the medical record, *TBI* traumatic brain injury, *ICP* intracranial pressure, *mRS* modified Rankin Score

^a^Mann-Whitney *U* test for numeric variables

^b^Fisher test for categorical variables; if more than 2 categories are given, Fisher test was performed for the 2 main categories

* *p* < 0.05

### Sex-related responder bias

Females of the non-responders (*n* = 68) and the QOLIBRI cohort (*n* = 33) differed regarding TBI etiology (*p* = 0.002), the elapsed time since TBI (*p* = 0.03), and the functional status at discharge from neurorehabilitation (*p* = 0.03), while the initial TBI severity (*p* = 0.04), a better functional outcome at discharge from neurorehabilitation (*p* < 0.0001), and less frequent tracheostomy (*p* = 0.02) were the only parameters that differed between males of the non-responders (*n* = 183) and the QOLIBRI cohort (*n* = 102) (Supplementary Table S1). TBI severity in males statistically differed between groups due to the high rate of missing data, i.e., 49.7% of male non-responders and 37.2% of males in the QOLIBRI cohort were not classified for their initial TBI severity (*p* = 0.04). Both groups, however, were functionally severely disabled when admitted to neurorehabilitation and did not differ between male (*p* = 0.65) and female (*p* = 0.78) non-responders and responders of the QOLIBRI cohort. Furthermore, female patients of the QOLIBRI cohort suffered on average 5.48 ± 0.53 years (mean ± SEM) from the brain injury, while the elapsed time since TBI was only 4.12 ± 0.3 years within the female non-responders (*p* = 0.03). Patients of both sexes within our QOLIBRI cohort regained better function from severe to slight disability during neurorehabilitation, while non-responders remained moderately disabled and needed support in daily activities though able to walk unassisted with a highly statistical difference between male (*p* < 0.0001) as well as a slight difference between female non-responders and responders (*p* = 0.03).

### Sex-related HRQoL up to 10 years after TBI

**Fig. 2:**
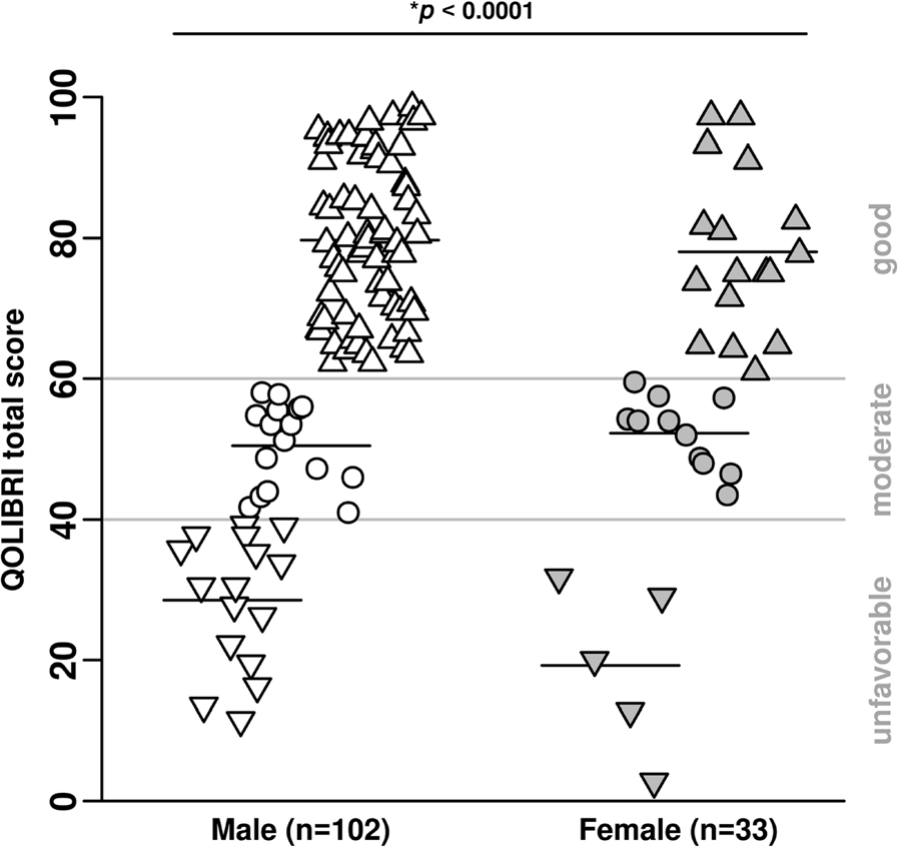
Sex-related quality of life in chronic TBI patients. A QOLIBRI total score ≥ 60 indicates good health-related quality of life (HRQoL), a score of 40–59 moderate and a score < 40 unfavorable HRQoL. Analyzing good, moderate, or unfavorable HRQoL revealed highly significant differences between male and female chronic TBI patients using the Kruskal-Wallis test (*p* < 0.0001). HRQoL, health-related quality of life; QOLIBRI, Quality of Life after Brain Injury; TBI, traumatic brain injury

**Fig. 3:**
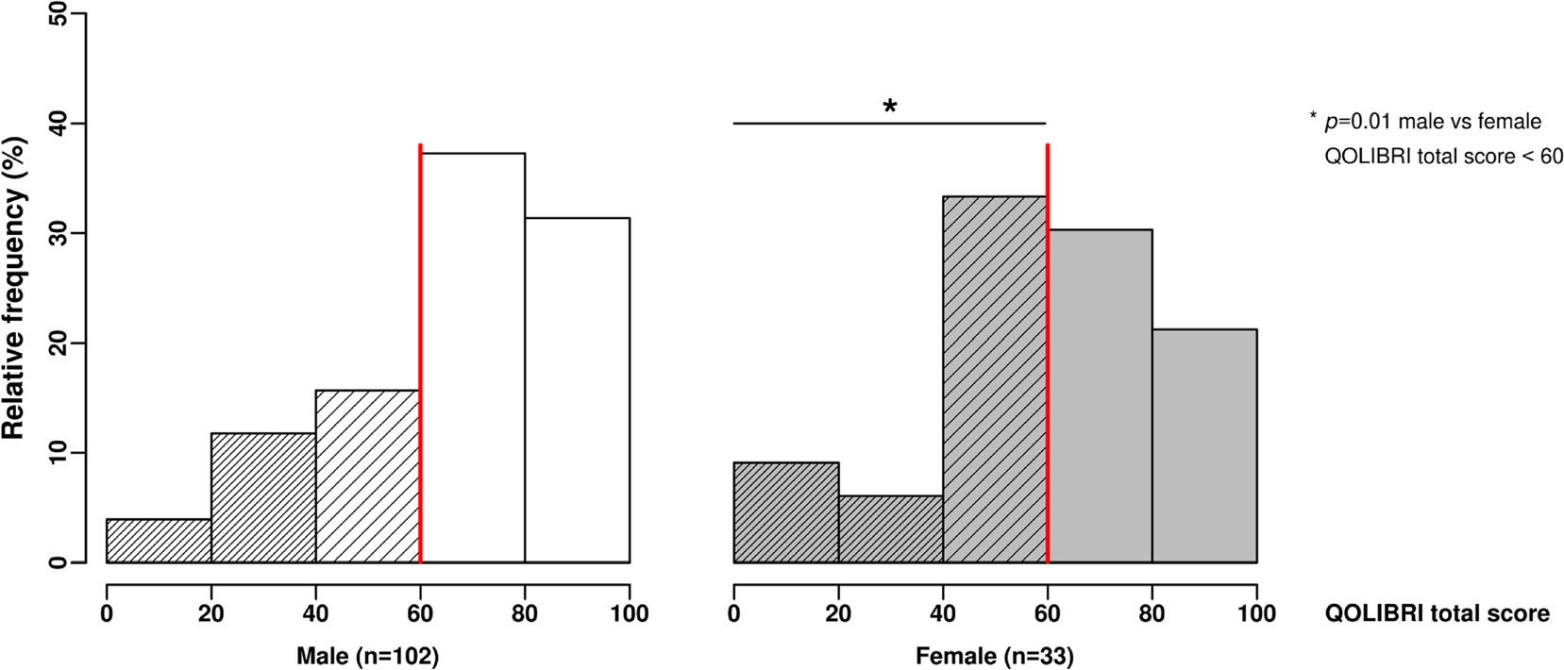
Females have an increased risk of psychiatric disorders after TBI. Seventeen percent more females (gray hatched bars) than males (white hatched bars) reported unsatisfactory HRQoL up to 10 years after TBI (*p* = 0.01) as indicated by the dichotomized analysis of relative frequencies distinguishing good (QOLIBRI total score ≥ 60) and unsatisfied HRQoL (QOLIBRI total score < 60) (red line) using the Fisher test. A total of 33% females and 16% males reported moderate HRQoL (QOLIBRI total score 40–59) with an increased risk of one posttraumatic psychiatric disorder. Females and males equally reported unfavorable HRQoL (QOLIBRI total score < 40), namely 16% of females (gray closely hatched bars) and 16% of males (white closely hatched bars), thereby having an increased risk of both psychiatric disorders after TBI. HRQoL, health-related quality of life; QOLIBRI, Quality of Life after Brain Injury; TBI, traumatic brain injury

Most of the chronic TBI patients of both sexes reported good HRQoL with a QOLIBRI total score equal or greater than 60 (*p* = 0.13) (Supplementary Fig. S1). Nevertheless, subgroup analysis revealed highly significant sex-related differences between good, moderate, and unfavorable HRQoL outcomes (*p* < 0.0001) (Fig. 2), as 68.6% of males reported good, 15.7% moderate, and 15.7% unfavorable HRQoL, while only 51.5% females stated good, 33.3% moderate, and 15.2% unfavorable HRQoL up to 10 years after TBI (Fig. 3). Thus, 17% more female than male patients were unsatisfied with their HRQoL during the chronic phase after TBI. Sex stratified and dichotomized analysis of good (QOLIBRI total score ≥ 60) versus unsatisfied HRQoL (QOLIBRI total score < 60), respectively, underlines the right shifted and thus better HRQoL long-term outcome in males after TBI (*p* = 0.01).

### Sex- and age-related HRQoL up to 10 years after TBI

**Fig. 4:**
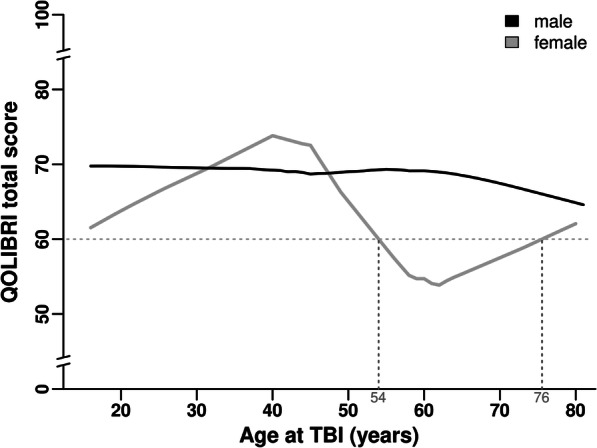
Age- and sex-related differences of HRQoL after TBI. Age at TBI matters for HRQoL in female, but not in male chronic TBI patients. Descriptive analysis revealed on average unsatisfied HRQoL (QOLIBRI total score < 60) of older females (solid gray line) aged 54 to 76 years at TBI, while males (black line) constantly reported good HRQoL over their entire adult lifespan using the LOWESS function. HRQoL, health-related quality of life; LOWESS, Locally Weighted Scatterplot Smoothing; QOLIBRI, Quality of Life after Brain Injury; TBI, traumatic brain injury

**Fig. 5:**
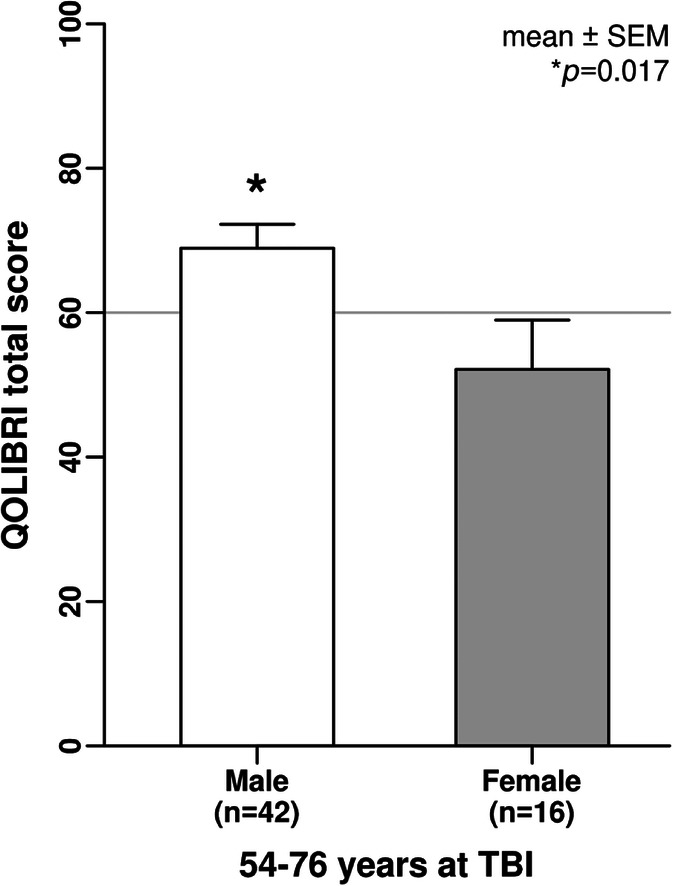
Older females with TBI suffer from moderate HRQoL. Females (gray bar) aged 54 to 76 years when having experienced a TBI reported a mean QOLIBRI total score of 52.1 ± 6.8 compared with males (white bar) with a mean of 68.9 ± 3.3 (*p* = 0.017). Data were normally distributed (D’Agostino-Pearson, omnibus K2) and compared with the unpaired t test. The effect size indicates a moderate to large effect with an eta squared of *η*^2^ = 0.1. HRQoL, health-related quality of life; QOLIBRI, Quality of Life after Brain Injury; TBI, traumatic brain injury

Over the adult lifespan, age at TBI matters for HRQoL in female but not in male patients of our QOLIBRI cohort. Females within their fifth to seventh decade of life stated on average moderate HRQoL with QOLIBRI total scores on average below 60, while males constantly reported good HRQoL with QOLIBRI total scores on average of 70 (Fig. 4). Post hoc sex- and age-related subgroup analysis uncovered worse HRQoL with a mean (± SEM) QOLIBRI total score of 52.1 ± 6.8 in 54–76-year-old females (*n* = 16) compared with males (*n* = 42) with a mean (± SEM) QOLIBRI total score of 68.9 ± 3.3 (*p* = 0.017) with a moderate to large effect size (*η*^2^ = 0.1) (Fig. 5). Demographic and basic characteristics of the 54–76-year-old male and female chronic TBI patients revealed statistical differences in terms of the initial TBI severity (*p* = 0.03), TBI etiology (*p* < 0.001), and a trend towards more females than males having received decompressive craniectomy (Supplementary Table S2).

### Self-reported cognition and physical problems after TBI during aging

**Fig. 6:**
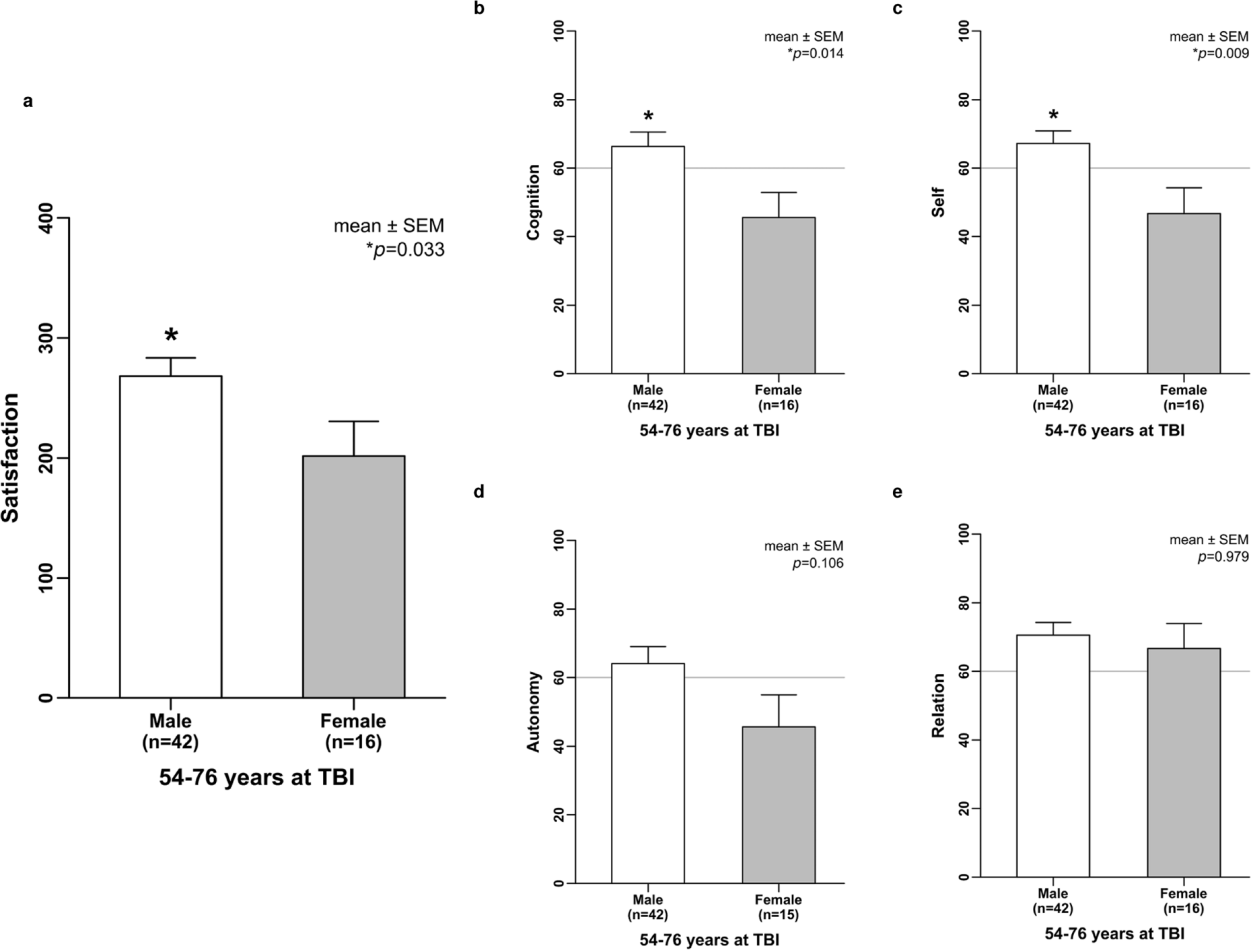

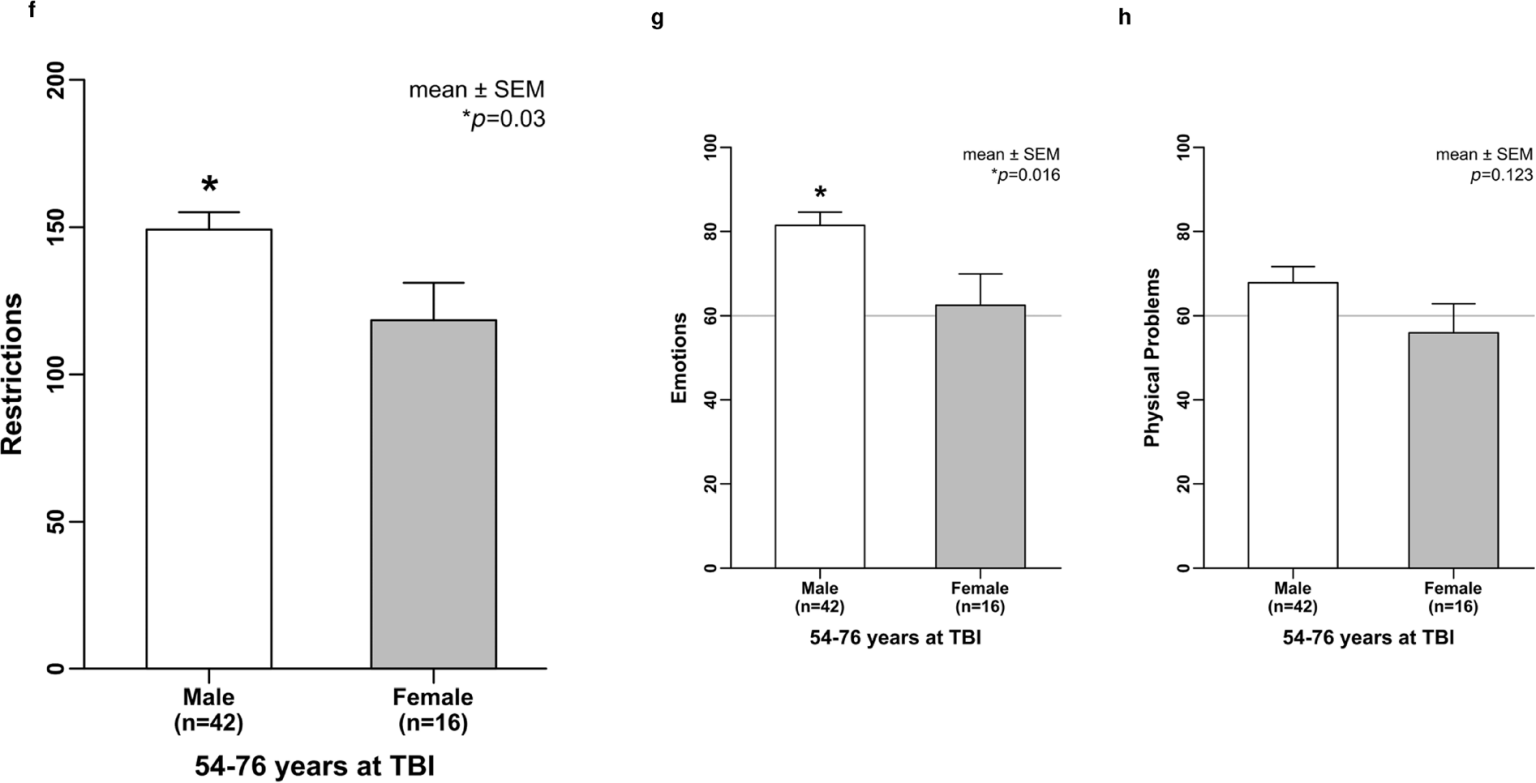
Cognition rather than physical problems hamper HRQoL when TBI hits the female brain during aging. Sex- and age-related difference was particularly obvious for the two key aspects of the QOLIBRI instrument, namely satisfaction (*p* = 0.033) and restriction (*p* = 0.03), as well as for the subscales of cognition (*p* = 0.014), self (*p* = 0.009), and emotions (*p* = 0.016), but not relevant in terms of daily life and autonomy, social relationships, and physical problems. Data of restrictions, autonomy, social relationships, and emotions were not normally distributed and thus analyzed by the Mann-Whitney *U* test. All other data were compared using the unpaired *t* test. HRQoL, health-related quality of life; QOLIBRI, Quality of Life after Brain Injury; TBI, traumatic brain injury

Older females aged 54 to 76 years at TBI reported unsatisfied HRQoL compared with males reporting good HRQoL up to 10 years after their brain impact. Within this age group of the 54–76-year-olds at TBI, sex-related difference of HRQoL was not only observed for the QOLIBRI total score **(**Fig.5**)** but also observed for the two key aspects of the QOLIBRI, namely satisfaction (*p* = 0.033) and restriction (*p* = 0.03), as well as for the subscales of cognition (*p* = 0.014), self (*p* = 0.009), and emotions (*p* = 0.016), while daily life and autonomy, social relationships, and physical problems did not differ between males and females (Fig. 6; Supplementary Table S3).

### TBI severity and sex-related HRQoL

Sex-related HRQoL measured by the QOLIBRI total score did not correlate with TBI severity based on the initial GCS as indicated in Table 2.

**Table 2:** Initial TBI severity does not correlate with HRQoL long-term outcome after TBI. Sex-related correlation analyses revealed no correlations between TBI severity based on the initial Glasgow Coma Scale (GCS) and HRQoL assessed by the QOLIBRI total score up to 10 years after the brain impact. However, the 54–76-year-old male patients showed a trend towards severer TBI correlating with worse HRQoL that needs further considerations in the future. GCS, Glasgow Coma Scale; QOLIBRI, Quality of Life after Brain Injury; TBI, traumatic brain injury

Correlation of HRQoL (QOLIBRI total score) with TBI severity (GCS)		
	Spearman’s rank correlation	*p* value
QOLIBRI cohort		
• Males with complete GCS data (*N* = 64)	− 0.2	0.2
• Females with complete GCS data (*N* = 20)	0.2	0.4
QOLIBRI subgroup aged 54–76 years at TBI		
• Males with complete GCS data (*N* = 24)	− 0.4	0.06
• Females with complete GCS data (*N* = 11)	0.2	0.6

## Discussion

In this cross-sectional study, we analyzed sex- and age-related HRQoL in 102 male and 33 female TBI patients aged 18 to 85 years up to 10 years after neurorehabilitation due to a TBI. Most chronic TBI patients of both sexes reported good HRQoL with a QOLIBRI total score equal or above 60. However, one-third of patients reported unsatisfied HRQoL, and 17% more females than males were moderately affected with an increased risk of one posttraumatic psychiatric disorder. Older females in their fifth to seventh decade of life reported significantly worse, while males constantly reported good HRQoL over their adult lifespan. When TBI hits the female brain during aging, particularly cognition, self-perception, and emotions rather than physical problems hampered HRQoL. Thus, these results stress the need to include quality of life for assessing TBI long-term outcome, thereby detecting neuropsychiatric sequelae after TBI early.

### The QOLIBRI cohort of chronic TBI patients under the sex lens

Our QOLIBRI cohort included 102 male and 33 female chronic TBI patients and is with a male to female ratio of 3:1 well in line with the literature [1, 6, 7, 25, 26]. This analysis covers a relatively and in particular small female sample size, but it is—to our knowledge the first analysis—that stratifies for age- and sex-related HRQoL over the adult lifespan including the oldest population up to 85 years. Furthermore, all patients received the most homogenous acute and neurorehabilitation treatment after brain injury, well-known to be relevant for patient’s outcome [27]. Males and females of the QOLIBRI cohort differed in terms of TBI severity classified by the initial GCS, but patients of both sexes—96% of males and 91% of females—were severely disabled when admitted to neurorehabilitation, suggesting a secondary deterioration after the initial classification of mildly brain injured, and thus rather a statistical than a content-relevant finding. While more females than males were initially classified severely injured, the data of their functional status shows rather the opposite, namely 9% of females compared with 4% of males were ambulatory. These data underline that TBI severity classified by the initial GCS is crucial during the emergency phase but less suitable for predicting TBI long-term outcome. Thus, a composite TBI classification is needed as aimed by the large TRACK-TBI and CENTER-TBI collaborations.

To draw proper conclusions from our data, it is important to evaluate whether males and females suffered comparable TBIs and were treated equally thereafter. In terms of TBI etiology, males suffered almost equally from traffic- or fall-related TBI while females suffered more often from a traffic-related TBI with a comparable average age at TBI of 47 years in both sexes. Although our current analysis cannot explain this difference, it needs further attention as the current literature attributes traffic-related TBI to the young and fall-related TBI to the elderly [1, 3]. Looking closer into our data, there were differences of TBI etiology in female non-responders and responders that need further consideration.

### Non-responders bias

The QOLIBRI cohort (N = 135) represents the natural occurrence of TBI with a male to female ratio of 3:1 and did not differ from non-responders (*N* = 251) of 183 males and 68 females with respect to sex distribution as previously published (*p* = 0.63) [20]. Therefore, a non-responder bias with respect to the male to female ratio within our QOLIBRI cohort is unlikely. Female non-responders suffered more often from a fall-related TBI than females of the QOLIBRI cohort, while TBI etiology did not differ between male non-responders and responders. One speculative hypothesis could be a higher frailty in female non-responders that might have interfered with study participation. Furthermore, there is evidence that fall-related TBI might predict dementia [8], possibly indicating here that elderly females who have experienced a fall-related TBI were not able to participate in our study due to a major disability or higher mortality rate; thus, most probably, our results even underestimate the larger female posttraumatic burden during aging.

The longer elapsed time since TBI in females of the QOLIBRI cohort compared with non-responders, namely on average 5.5 and 4 years since TBI, respectively, statistically differed but is not likely impacting results given that HRQoL is usually worse during the first year after TBI and remains quite stable afterwards [20, 28]. Male patients of the QOLIBRI cohort received less frequently a tracheostomy than male non-responders—a difference which was not seen between (i) the female responders and non-responders, (ii) the male and female patients of the QOLIBRI cohort, and (iii) the subgroup of the 54–56-year-old patients, thus indicating a slight non-responder bias in male patients that most probably does not influence the presented results. Furthermore, males and females of the QOLIBRI cohort regained better function from severe to slight disability during neurorehabilitation, while non-responders remained moderately disabled and needed support in daily activities although able to walk unassisted; thus, a non-responder bias cannot fully be excluded.

### Sex-related HRQoL and the female risk of posttraumatic psychiatric disorders

Sex is not a predictor for HRQoL up to 10 years after neurorehabilitation in initially severely disabled TBI patients [20]. However, 69% of males and only 52% females were satisfied with their HRQoL; thus, 17% more females were at risk of one posttraumatic psychiatric disorder, particularly depression or anxiety. Unfavorable HRQoL outcome, i.e., a QOLIBRI total score below 40, was equally distributed among male and female chronic TBI patients with 16% of both sexes having an increased risk of two psychiatric disorders. To date, sex-stratified data on posttraumatic depression and anxiety is scarce and controversial. In a large prospective study, 56% of 774 mild, moderate, or severe TBI patients had depression at 3 months after the brain impact of which 22% had a pre-existing history of psychiatric disorder with female sex as an independent predictor of depression [29]. In contrast, Dikmen and colleagues found males and lower education as risk factors of posttraumatic depression while age and TBI severity did not influence psychiatric outcome [30]. Further studies elucidated older males and pre-existing neuropsychiatric disorders as risk factors for posttraumatic depression [31, 32], while other studies did not find or did not stratify for sex-related differences regarding posttraumatic depression or anxiety [33–37]. Thus, our results pinpoint the need for psychiatric diagnostics to minimize the burden of treatable posttraumatic psychiatric disorders that might particularly affect females’ HRQoL after TBI.

### Age at TBI matters for HRQoL in female chronic TBI patients

Older females who experienced TBI within their fifth to seventh decade of life, i.e., between 54 and 76 years of age, were moderately affected when assessing their HRQoL having an increased risk of one psychiatric disorder in our QOLIBRI cohort—a relevant finding that was seen neither in our male TBI patients nor in the aged-matched healthy German population using the SF-36 instrument as described by Ellert and Kurth [38]. To date, there are still no norm values available for the QOLIBRI instrument but good correlations between the QOLIBRI total score and the generic SF-36 instrument have been described [12], and therefore, the latter study results are currently the best available to compare HRQoL of the healthy German population to patients suffering from TBI. To our knowledge, our main finding, namely the decreased HRQoL in elderly females after TBI, was not described previously and needs further consideration in future studies. This is particularly relevant as our results most probably underestimate the female posttraumatic burden when experiencing a TBI during midlife or aging due to the non-responder bias as reflected by lack of fall-related female chronic TBI patients with potentially increased mortality, frailty, or dementia [8].

Demographic and basic characteristics of the 54–76-year-old male and female chronic TBI patients revealed statistical differences in terms of the initial TBI severity, TBI etiology, and a trend towards more females having received decompressive craniectomy than males. As functional status at admission to and discharge from neurorehabilitation did not differ between males and females, we interpret TBI severity with males potentially having had milder injuries due to the initial GCS of minor relevance for the current long-term outcome results. As mentioned above, the impact of TBI etiology on HRQoL in the long run needs to be investigated in larger cohorts. Regarding decompressive craniectomy after TBI, we previously showed that having received decompressive craniectomy was associated with better HRQoL in the elderly compared with patients that have not received this neurosurgical intervention [21]; thus, our results even underestimate the reduced quality of life in older females.

### Cognition rather than physical problems hampers HRQoL when TBI hits the female brain during aging

The analysis of subscales revealed significant age- and sex-related differences in terms of cognition, self-perception, and emotions, while daily life and autonomy, social relationships, and physical problems did not differ between males and females aged 54–76 years at TBI. This is a most relevant finding as neurorehabilitation primarily focus on physical rehabilitation aiming to regain independence in daily activities. It is known that TBI patients have a lifelong increased risk of posttraumatic anxiety that increases with age and often occurs with comorbid depression [36]. The latter finding and our results highlight the importance of neuropsychiatric long-term monitoring after TBI to elucidate posttraumatic psychiatric sequelae that are treatable and impact particularly older females’ well-being after TBI.

### TBI severity and sex-related HRQoL

TBI severity did not correlate with sex-related HRQoL up to 10 years after the brain impact; particularly, no correlations were found for males and females over the entire adult life span and for the subgroup of the 54–76-year-olds at TBI of our QOLIBRI cohort. Previously, we reported that the initial TBI severity is a slight contributor but not a strong predictor of HRQoL in our cohort [20], and these data are well in line with the German QOLIBRI validation study of 172 TBI patients [12]. In contrast, the Finnish sample of the international QOLIBRI validation study found a negative correlation between TBI severity and HRQoL in 143 patients who had received intensive rehabilitation during 1993–2006 indicating lower HRQoL in patients having experienced milder brain injuries [39]. However, current evidence is still not fully clear yet, and thus, we underline the need for a better TBI severity classification beyond the initial GCS for TBI outcome prediction and to include HRQoL assessment in clinical practice.

### Current knowledge and gaps in sex- and gender-related TBI research

TBI studies provide controversial results on sex-related outcome, and sex-stratified approaches are underutilized in TBI research and clinical practice [6, 7, 40, 41]. One major confounder in sex-related TBI outcome is that females consistently report symptoms more often during the first 3 to 6 months after mild to moderate TBI [42–44], but this does not factor into the current results as the majority of our cohort experienced a severe TBI and no females were included within 6 months after TBI. However, the arising question is why female aging might be a risk factor for poor HRQoL with an increased risk of posttraumatic psychiatric disorders? Interestingly, female myelin seems to be more vulnerable and susceptible to damage during the perimenopausal transition in experimental and clinical studies [45, 46], and thus, further research into myelin vulnerability during aging in females is necessary. Furthermore, decreased glucose metabolism in females during aging seems to be one potential contributor to the increased female risk of dementia as recently found in animal studies [47] and needs consideration in clinical TBI research. Additionally, brain morphology seems to differ between sexes with males having larger brain volumes and larger white matter volumes than females [48]. Thus, males might be able to compensate comparable injuries better. Moreover, the lack of female sex hormones might be of relevance for the impeded HRQoL during aging in females as previous experimental TBI research uncovered estrogen and progesterone to be neuroprotective by downregulating cerebral inflammation and glutamatergic excitation [49–51]. Finally, socio-economic factors such as financial independence, differences in caregiving, and awareness of disability might be of relevance for the less favorable HRQoL outcome in females during aging. One often neglected factor is that females live longer than males, and therefore, male TBI victims more frequently benefit from lifelong female caregivers than vice versa.

### Limitations and generalizability

Several limitations of our findings warrant discussion. Firstly, the female sample size is small. However, this is the first study to investigate the impact of sex on long-term HRQoL after TBI in an aging population that has received a uniform acute and rehabilitative treatment. Our results suggest that there is a need for stratifying age, sex- and gender-related factors after TBI as these factors can impact on outcome. Secondly, TBI etiology differs between males and females with significantly fewer participating females suffering from fall-related TBI and this sex-related difference even increased during aging in our QOLIBRI cohort. Why females who suffered from a TBI due to a fall were less likely to participate in this study remains unclear, but our results even underestimate the females’ long-term burden after TBI. Thus, we suggest incorporating sex- and age-related analysis of comorbidity, frailty, and mortality with respect to TBI etiology in future TBI outcome research. Third, functional outcome was not routinely assessed using the GOSE but by the mRS during neurorehabilitation at the Schoen Rehabilitation Center. The mRS at discharge from neurorehabilitation did not differ between male and female patients of the QOLIBRI cohort. Hence, the assessment of HRQoL yields additional and therapeutically relevant data as compared with traditional outcome scores. Fourth, why midlife and older females experience less good HRQoL than aged-matched males in the long run after TBI needs further investigation with age-, sex-, and gender-related analyses including clinical parameters, environmental factors, as well as fluid and neuroimaging biomarkers that might help to strengthen the link between psychiatric disorders and the risk of posttraumatic cognitive burden or even dementia [52–54].

### Generalizability and future perspectives

Regarding the generalizability of this study, most of the demographic and basic characteristics were representative of the entire TBI cohort of 439 patients. Therefore, results are probably at least generalizable to industrialized countries providing comparable acute and rehabilitative care after TBI.

Our results uncover the significance of assessing HRQoL in the long run after TBI and thus should be a valuable tool in clinical routine after TBI. Furthermore, we emphasize the importance of neuropsychiatric follow-ups especially in females who have experienced TBI during aging. If restricted HRQoL long-term outcome might be a first link to an underlying age- and sex-related brain vulnerability or neuroinflammatory response with the risk of posttraumatic dementia needs to be tackled in the future.

## Conclusion

Experiencing TBI during aging does not influence HRQoL outcome in males but females suggesting that female brains cope less well with a traumatic injury during aging. Therefore, older females particularly need long-term follow-up assessments after TBI to detect and treat neuropsychiatric sequels that restrict their quality of life. Further investigations are necessary to uncover the mechanisms of this so far unknown phenomenon.

## Electronic supplementary material

ESM 1 (PDF 52 kb)

ESM 2 (PDF 50.0 kb)

ESM 3 (PDF 136 kb)

## Data Availability

The data sets of the current study are available from the corresponding author on reasonable request. All data are included in this article and its supplementary material.

## Abbreviations

CENTER-TBI: Collaborative European NeuroTrauma Effectiveness Research in traumatic brain injury
DALY: Disability-adjusted life years
GCS: Glasgow Coma Scale
GOSE: Extended Glasgow Outcome Scale
HRQoL: Health-related quality of life
ICP: Intracranial pressure
LOWESS: Locally Weighted Scatterplot Smoothing is used to create a smooth line through a scatter plot to visualize relationships between variables and foresee trends
mRS: Modified Rankin Scale
QOLIBRI: Quality of Life after Brain Injury: the QOLIBRI instrument is a health-related, disease specific and internationally validated instrument to assess health-related quality of life in individuals following brain injury
SEM: Standard error of the mean
SF-36: Short Form (36) Health Survey: a 36-item patient-reported questionnaire regarding health status, used in medical outcome studies
TBI: Traumatic brain injury
TRACK-TBI: Transforming Research and Clinical Knowledge in Traumatic Brain Injury
WHO: World Health Organization
