# Supplementary figures and images for: Quality of life after traumatic brain injury: a cross-sectional analysis uncovers age- and sex-related differences over the adult life span
Source: GeroScience. 2020 Oct 17;43(1):263–78. doi: 10.1007/s11357-020-00273-2 (PMC8050174; doi:10.1007/s11357-020-00273-2)

Supplementary Figure S1

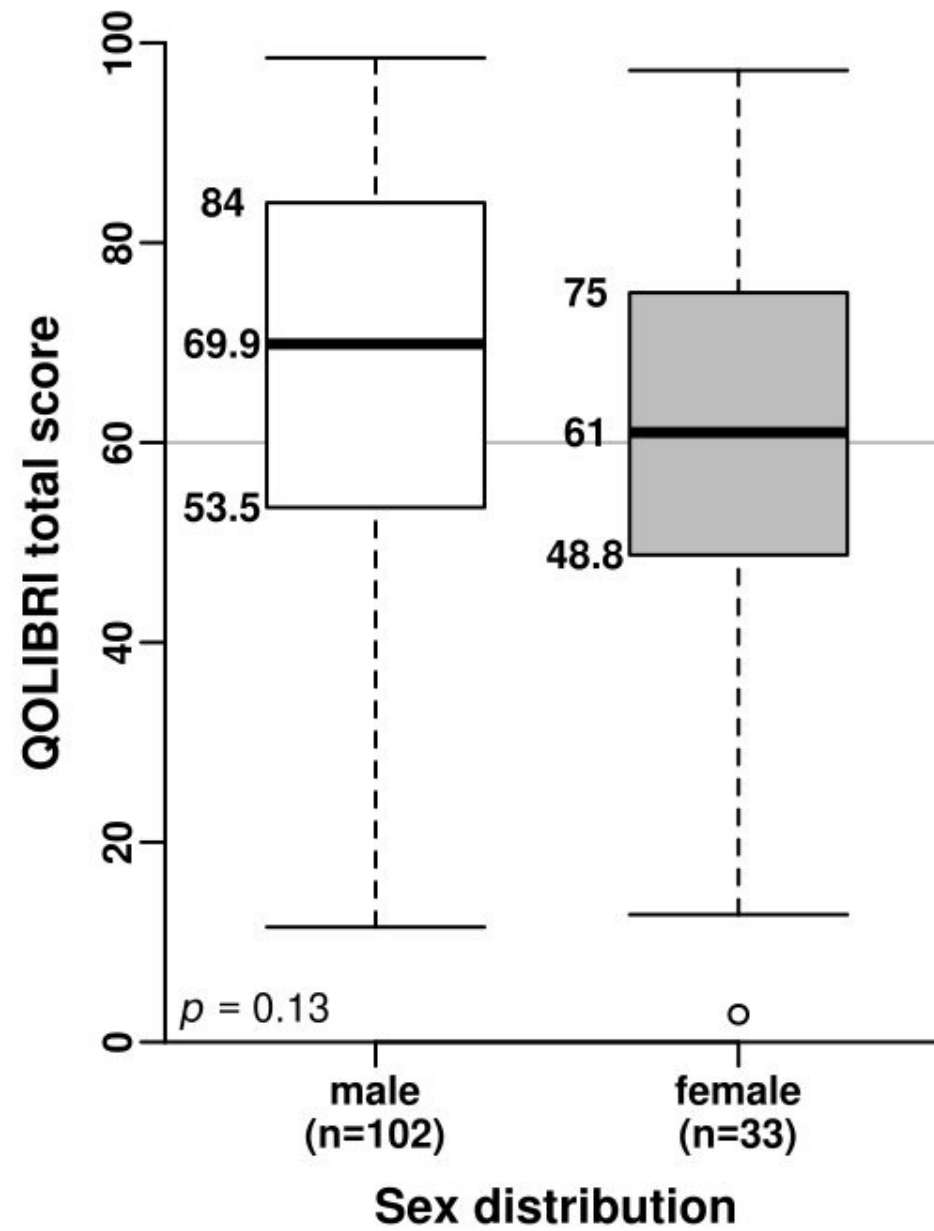

Supplement: Supplementary file 1 — (PDF 52 kb) [file 11357_2020_273_MOESM1_ESM.pdf]

Supplementary Figure S2

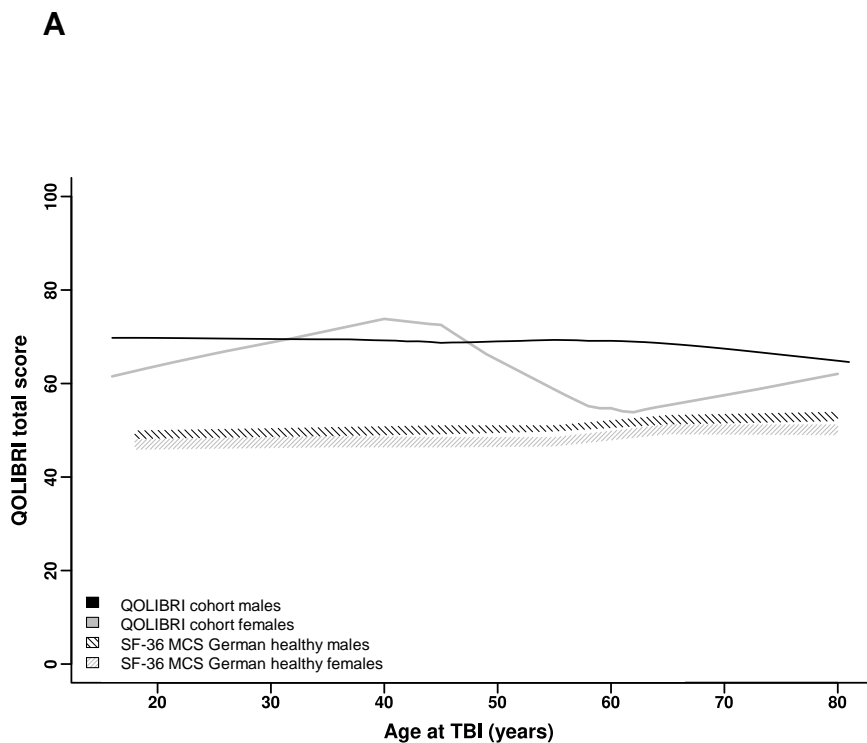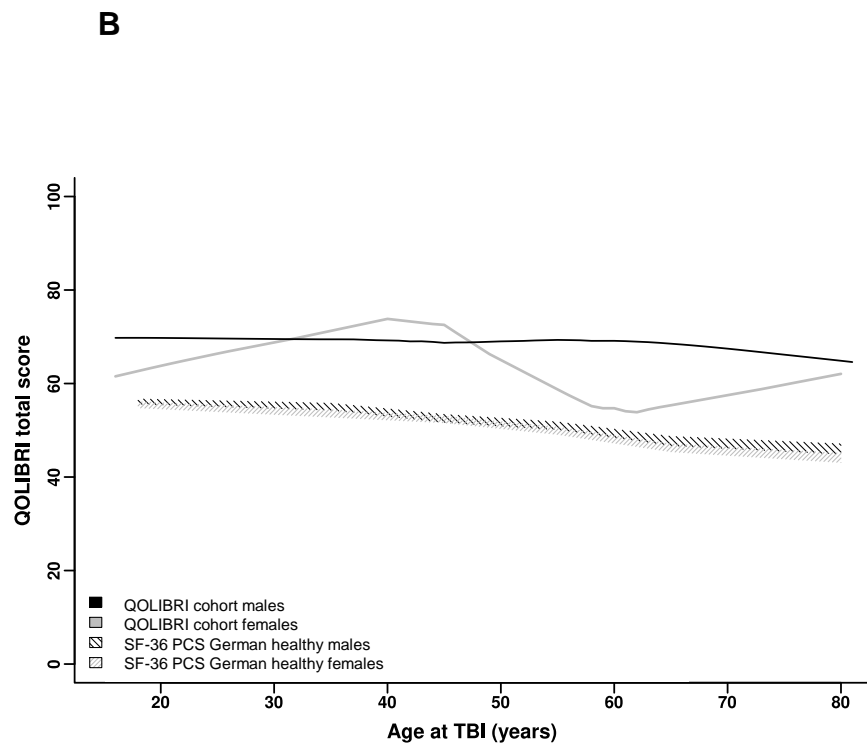

Supplement: Supplementary file 2 — (PDF 50.0 kb) [file 11357_2020_273_MOESM2_ESM.pdf]
