# Supplementary material for: Quality of life after traumatic brain injury: a cross-sectional analysis uncovers age- and sex-related differences over the adult life span
Source: GeroScience. 2020 Oct 17;43(1):263–78. doi: 10.1007/s11357-020-00273-2 (PMC8050174; doi:10.1007/s11357-020-00273-2)
Supplement: Supplementary file 3 — (PDF 136 kb) [file 11357_2020_273_MOESM3_ESM.pdf]

Supplementary Table S1

	Non-responder	QOLIBRI cohort		Non-responder	QOLIBRI cohort	
	male n=183	male n=102	*p*-value	Female n=68	female n=33	*p*-Value
**TBI severity** mild/ moderate/ severe/ n.s. (%) [a]	9.3/6/35/49.7	16.7/13.7/32.4/37.2	0.04*	2.9/5.9/25/66.2	3/12.1/45.5/39.4	0.9
**TBI etiology** traffic accidents/ falls/ others (%) [b]	45.9/44.3/9.8	43.1/46.1/10.8	0.79	36.8/54.4/8.8	72.7/24.2/3.1	0.002**
**Age at TBI** (mean ± SEM) [a]	49.67 ± 1.52	47.41 ± 1.98	0.35	54.69 ± 2.63	47.76 ± 3.36	0.07
**Age at survey** (mean ± SEM) [a]	55.3 ± 1.54	53.08 ± 1.92	0.38	58.87 ± 2.62	53.24 ± 3.19	0.13
**Time since TBI** (mean ± SEM) [‡]	5.56 ± 0.23	5.71 ± 0.3	0.75	4.12 ± 0.3	5.48 ± 0.53	0.03*
**Decompressive craniectomy** (%) [b]	32.2	24.5	0.18	30.9	36.4	0.65
**ICP monitoring or permanent shunt device** (%) [b]	64.5	55.9	0.16	54.4	60.6	0.67
**Tracheostomy** (%) [b]	68.9	54.9	0.02	47.1	51.5	0.83
**Time to onset of neurorehabilitation** [a] (days, mean ± SEM)	30.5 ± 3.05	26.78 ± 2.89	0.51	27.78 ± 3.85	25.61 ± 3.22	0.59
**Duration of neurorehabilitation** [a] (days, mean ± SEM)	52.94 ± 3.71	36.68 ± 2.81	0.05	48.76 ± 7.11	46.09 ± 7.81	0.88
**Functional status at admission** (mobile (mRS 0- 3)/ immobile (mRS 4-5)/ n.c. (%)) [b]	8.2/91.8/0	3.9/95.1/1	0.22	17.6/82.4/0	9.1/90.9/0	0.37
(mean ± SEM) [a]	4.61 ± 0.07	4.65 ± 0.07	0.65	4.31 ± 0.15	4.46 ± 0.18	0.78
**Functional status at discharge** (mobile (mRS 0- 3)/ immobile (mRS 4-5)/ n.c. (%)) [b]	59/41/0	82.4/17.6/0	<0.001**	60.3/39.7/0	75.8/24.2/0	0.18
(mean ± SEM) [a]	3.17 ± 0.11	2.23 ± 0.12	<0.0001***	3.01 ± 0.2	2.3 ± 0.23	0.03*

[a] Mann-Whitney-U-Test for numeric variables
[b] Fisher-Test for categorical variables; if more than 2 categories are given, Fisher-Test was performed for the 2 main categories
n.c.: not classified in the medical record; n.s.: not specified in the medical record; TBI: traumatic brain injury; ICP: intracranial pressure; mRS: modified Rankin Score

Supplementary Table S2

	QOLIBRI cohort Subgroup of 54-76-year-olds		
	**male n=42**	**female n=16**	***p*-value**
**TBI severity** mild/ moderate/ severe/ n.s. (%) [a]	28.6/14.3/14.3/42.8	6.2/25/37.5/31.3	0.03*
**TBI etiology** traffic accidents/ falls/ others (%) [b]	31/61.9/7.1	81.2/12.5/6.3	<0.001*
**Age at TBI** (mean ± SEM) [a]	65.38 ± 1.08	62.5 ± 1.67	0.22
**Age at survey** (mean ± SEM) [a]	70.55 ± 1.16	67.12 ± 1.67	0.13
**Time since TBI** (mean ± SEM) [‡]	5.31 ± 0.45	4.69 ± 0.69	0.42
**Decompressive craniectomy** (%) [b]	19	43.8	0.09
**ICP monitoring or permanent shunt device** (%) [b]	50	62.5	0.56
**Tracheostomy** (%) [b]	59.5	56.2	1
**Time to onset of neurorehabilitation** [a] (days, mean ± SEM)	26.07 ± 2.81	27.5 ± 5.68	0.97
**Duration of neurorehabilitation** [a] (days, mean ± SEM)	40.48 ± 4.65	40.12 ± 8.47	0.73
**Functional status at admission**			
(mobile (mRS 0- 3)/ immobile (mRS 4-5) (%)) [b]	7.1/92.9	6.2/93.8	1
(mean ± SEM) [a]	4.52 ± 0.15	4.69 ± 0.15	0.77
**Functional status at discharge**			
(mobile (mRS 0- 3)/ immobile (mRS 4-5) (%)) [b]	78.6/21.4	75/25	0.74
(mean ± SEM) [a]	2.48 ± 0.18	2.44 ± 0.36	0.74

[a] Mann-Whitney-U-Test for numeric variables
[b] Fisher-Test for categorical variables; if more than 2 categories are given, Fisher-Test was performed for the 2 main categories
n.s.: not specified in the medical record; TBI: traumatic brain injury; ICP: intracranial pressure; mRS: modified Rankin Score

Supplementary Table S3

Health-related quality of life in the subgroup of 54-76-year-olds at TBI<br>Mean ± SEM			
**QOLIBRI item**	**Male**<br>**n=42**	**Female**<br>**n=16**	***P*-value**
QOLIBRI total score [b]	68.9 ± 3.3	52.1 ± 6.8	0.017*
Satisfaction [a]	268.2 ± 15.2	201.7 ± 28.9	0.033*
Cognition [a]	66.3 ± 4.2	45.6 ± 7.3	0.014*
Self [a]	67.2 ± 3.7	46.7 ± 7.5	0.009**
Daily life & autonomy [b]	64.1 ± 5.0	45.7 ± 9.3 [c]	0.106
Social relationships [b]	70.6 ± 3.7	66.7 ± 7.3	0.979
Restrictions [b]	149.2 ± 5.9	118.4 ± 12.6	0.03*
Emotions [b]	81.4 ± 3.2	62.5 ± 7.4	0.016*
Physical problems [a]	67.8 ± 3.9	55.9 ± 6.9	0.123

Key aspects satisfaction (score 0-400) and restrictions (score 0-200) are sum scores of subcategories as indicated
[a] Normal distributed data according to D'Agostino-Pearson, omnibus K2; unpaired t-test
[b] Mann-Whitney-*U* test for non-parametric data
[C] For daily life & autonomy 15 females gave information
